# Impact of safety warnings for fluoroquinolones on prescribing behaviour. Results of a cohort study with outpatient routine data

**DOI:** 10.1007/s15010-020-01549-7

**Published:** 2020-11-30

**Authors:** Ulrike Georgi, Falko Tesch, Jochen Schmitt, Katja de With

**Affiliations:** 1 Pharmacy Service of Clinical Center, Flemmingstrasse 2, 09116 Chemnitz, Germany; 2 Center for Evidence-Based Healthcare, University Hospital and Medical Faculty Carl Gustav Carus, TU Dresden, Dresden, Germany; 3 Division of Infectious Diseases, University Hospital Carl Gustav Carus, TU Dresden, Dresden, Germany

**Keywords:** Antibiotic use, Cohort studies, Fluoroquinolones, Patient safety, Practice patterns, Pharmacovigilance

## Abstract

**Purpose:**

The need for drug-related safety warnings is undisputed, but their impact on prescribing behaviour is not always clear. Safety warnings usually do not contain therapeutic alternatives. Based on German outpatient routine healthcare data, our cohort study investigated the impact of three warnings for fluoroquinolones on prescribing behaviour.

**Methods:**

Structural breaks were estimated in a time-series analysis (2005–2014) of 184,134 first antibiotic prescriptions for patients (≥ 18 years) diagnosed with community-acquired pneumonia (CAP), acute bacterial sinusitis (ABS), or acute exacerbation of chronic bronchitis (AECB). Subsequently, risk factors for patients’ before/after safety warnings presented as risk ratios (RR) were estimated by Poisson regression.

**Results:**

Following the 2008 warning for moxifloxacin, the RR of being prescribed moxifloxacin was reduced by 56% (95% CI 0.41–0.47; *p* < 0.001) for CAP, by 65% (95% CI 0.32–0.39; *p* < 0.001) for ABS, by 57% (95% CI 0.41–0.45; *p* < 0.001) for AECB. After the 2012 warning for levofloxacin, the RR of being prescribed levofloxacin was reduced by 31% (95% CI 0.64–0.74; *p* < 0.001) for CAP, by 14% (95% CI 0.77–0.96; *p* = 0.007) for ABS, by 27% (95% CI 0.69–0.77; *p* < 0.001) for AECB. We noticed a prescription-switch to other antibiotics which was not in line with the national guideline recommendations. The warning for moxifloxacin 2009 had no impact on prescribing behaviour.

**Conclusion:**

This study observed an impact on prescribing behaviour in response to regulatory safety warnings for two out of three warnings. Information on therapeutic alternatives should be a part of any safety warning to encourage the intended changes in prescribing behaviour.

**Electronic supplementary material:**

The online version of this article (10.1007/s15010-020-01549-7) contains supplementary material, which is available to authorized users.

## Introduction

Written safety warnings on human drugs addressed to health care professionals (e.g., physicians and pharmacists) intend to minimise the risk of drug therapy. The need for drug-related safety warnings is therefore undisputed and three systematic reviews [1–3] investigated their impact on prescribing behaviour. Many studies included in these reviews described the impact of safety warnings contradictorily. Furthermore, most of the studies were conducted in the United States or Canada, some in Europe and a few in other countries. In Germany, the impact of Dear Doctor Letters (“Rote-Hand-Brief”) on prescribing behaviour was only investigated partially [4].

In Europe, the Pharmacovigilance Risk Assessment Committee (PRAC) of the European Medicines Agency (EMA) is responsible for initiating risk assessment procedures and recommends pertinent safety information for human drugs [5]. The EMA closely cooperates with the national competent authorities, e.g., in Germany with the Federal Institute for Drugs and Medical Devices (BfArM). German pharmaceutical companies are legally obliged to make therapy-relevant changes available to healthcare professionals. The BfArM determines the form in which the changes are to be communicated, e.g., as Dear Doctor Letters (“Rote-Hand-Brief”).

Germany published three Dear Doctor Letters on fluoroquinolones in 2008, 2009 and 2012. Fluoroquinolones are orally or intravenously administered broad-spectrum antibiotics. National guidelines valid at the time of analysis, e.g., on community-acquired pneumonia (CAP), recommended moxifloxacin and levofloxacin to patients with CAP and additionally risk factors only if no alternative treatment was available [6, 7].

Nevertheless, fluoroquinolone consumption increased over years and became popular in treating even less severe infections in primary care [8]. In February 2008, a moxifloxacin-Dear Doctor Letter informed about new serious side effects (fulminant hepatitis, potentially life-threatening skin reactions) but without any indication restrictions [9]. In July 2008, the EMA´s Committee for Medicinal Products for Human Use (CHMP) decided to restrict the indications of oral moxifloxacin regarding CAP, acute bacterial sinusitis (ABS) and acute exacerbation of chronic bronchitis (AECB) [10], followed by another Dear Doctor Letter for German healthcare professionals in January 2009 [11]. The Dear Doctor Letter pointed out that in the treatment of CAP oral moxifloxacin-containing medicines should only be prescribed if no other antibiotics can be used, and in the treatment of ABS or AECB if other antibiotics cannot be used or have failed. Furthermore, side effects such as arrhythmias were supplemented to the product information.

In September 2012, based on the CHMP decision, a Dear Doctor Letter [12] restricted the indications for oral and intravenous levofloxacin in Germany. The diagnoses CAP, ABS and AECB were restricted in the same way as in the Dear Doctor Letter for moxifloxacin [11]. In addition, as for moxifloxacin, the product information was supplemented by side effects such as arrhythmias.

We report results of a cohort study to investigate if the Dear Doctor Letters on moxifloxacin (02/2008, 01/2009) and levofloxacin (09/2012) led to an impact on prescribing behaviour in adult outpatients in the federal state of Saxony. Primarily, the study was to answer the following questions: (1) Has the number of first prescriptions of moxifloxacin and levofloxacin for CAP, ABS and AECB been reduced in accordance with the Dear Doctor Letters? (2) Was there a prescription-switch to other antibiotics (in line with the national guideline recommendations)? Secondarily, we investigated the influence of the additional diagnoses of an arrhythmia or a pre-existing allergy (in case of a penicillin allergy) on the prescribing behaviour.

## Methods

### Data source

Almost 90% of Germany’s population (~ 82 million inhabitants) are covered by statutory health insurances of which the AOK (Allgemeine Ortskrankenkasse) is one of the biggest in Germany. Saxony is a federal state in eastern Germany with about 4 million inhabitants. The largest statutory health insurance, the AOK PLUS, covers approximately half of the general population in Saxony, i.e., ~ 2 million inhabitants [13]. This cohort study was based on pseudonymised routine healthcare data from the AOK PLUS.

According to § 295 and § 300 section 1 of the German Social Security Code V, the database holds information such as outpatient care data in regard to diagnoses (International Statistical Classification of Diseases, Tenth Revision [ICD-10]), procedures and prescriptions (Anatomical Therapeutic Chemical [WHO-ATC]) and socio-demographic information.

### Ethics

The study protocol was approved by the Ethics Committee of the Medical Faculty of the TU Dresden, Germany (no. EK 57022017).

### Study cohort, variables and measures

Prescription data for adult patients (≥ 18 years of age, covariates age and sex) with a documented diagnosis of an infection for the period from 2005 to 2014 (40 quarters) with the WHO-ATC classification codes J01CR02 (amoxicillin and beta-lactamase inhibitor [BLI]), J01CR04 (sultamicillin), J01DC02 (cefuroxime), J01DD13 (cefpodoxime), J01MA12 (levofloxacin) and J01MA14 (moxifloxacin) were analysed. According to the German guidelines, amoxicillin/BLI or sultamicillin were recommended for the first line treatment of patients with CAP and additional risk factors, while levofloxacin, moxifloxacin, cefpodoxime and cefuroxime were mentioned as alternatives [6, 7]. The same antibiotics were recommended in the German guidelines for patients with ACEB and severe pulmonary function impairment [6, 7] as well as for patients with severe ABS [14]. Furthermore, all other prescribed substances with the WHO-ATC code J01 (except J01CR02, J01CR04, J01DC02, J01DD13, J01MA12 and J01MA14) for patients diagnosed with the ICD-10 codes defined below were subsumed under the group “other antibiotics”. In addition, the study was limited to the number of first prescriptions of the antibiotics dispensed. Possible changes to the ATC codes over time have been checked for each year [15].

The following diagnoses according to ICD-10 codes [16] were included: J01 (ABS), J13-J16 and J18 (CAP), J44 (AECB), I44-49 (arrhythmia), L27 excl. L27.2 and Y57 and Z88 excl. Z88.4-88.8 (pre-existing allergy). In contrast to other routine healthcare data [17], outpatient diagnoses in Germany are available quarterly only. The prescription data are available on the exact day. According to Schulz et al. [18], data were limited for patients who had only one documented diagnosis of an infection and no further documented infection during a quarter. To examine the impact of each Dear Doctor Letter on the prescribing behaviour, eight quarters before and eight quarters after the intervention year (publication year of the Dear Doctor Letter) were analysed. Therefore, the year of the respective intervention was not included in the analysis.

Exclusion criteria for patients were pregnancy, birth and puerperium (ICD 10 code group O), other bacterial infections (ICD 10 code A 00-A 79), documented infection without a prescription and documented suspected diagnosis of an infection. Furthermore, the antibiotic had to be prescribed by the same physician who also documented the infection; otherwise, we excluded the data record.

### Statistical analyses

The data analysis was carried out on two levels. First, at the individual patient level, risk factors presented as risk ratios (RR) for an antibiotic prescription eight quarters before and eight quarters after the intervention year were estimated by Poisson regression with robust standard errors [19]. At the second level, the prescription data were clustered by disease groups and quarters. Here we followed the approach by Bai and Perron [20], which has been implemented into the R package “strucchange” by Zeileis et al. [21, 22]. The idea was to segment the data multiple times into different long periods and to estimate each time how well the data are represented by the segmented regressions according to the Bayesian information criteria. This was used to determine how many change points were likely to be present in the time series and when they occurred.

## Results

A total of 226,541 first prescriptions dispensed met the eligibility criteria of the defined study population. According to the defined analysis criteria (8 quarters before and after the intervention year) 184,134 prescriptions of moxifloxacin, levofloxacin, amoxicillin and beta-lactamase inhibitor, sultamicillin, cefuroxime, cefpodoxime and the antibiotics subsumed under the “other antibiotics” group were included in the investigation. The mean age of the treated patients was between 67 and 70 years for CAP and AECB, while it was about 44 years for ABS. CAP and AECB were documented almost equally frequently in both men and women. Nearly two thirds of the patients with diagnosed ABS were female. The study population is also described in Table 1.

**Table 1:** Study population of the pre- and postintervention periods for two Dear Doctor Letters

	Dear Doctor Letter for moxifloxacin (02/2008)		Dear Doctor Letter for levofloxacin (09/2012)	
	Preintervention period 2006/2007	Postintervention period 2009/2010	Preintervention period 2010/2011	Postintervention period 2013/2014
Community-acquired pneumonia (CAP)				
Patients with antibiotic prescription	7189	8415	8256	8280
Mean age [years] (SD)	67.76 (18.80)	68.42 (18.91)	67.84 (19.20)	69.05 (18.47)
Women *n* (%)	3969 (55.21)	4576 (54.38)	4440 (53.78)	4387 (52.98)
Arrhythmias *n* (%)	1053 (14.65)	1470 (17.47)	1540 (18.65)	1709 (20.64)
Pre-existing allergy *n* (%)	26 (0.36)	39 (0.46)	45 (0.55)	48 (0.58)
Acute bacterial sinusitis (ABS)				
Patients with antibiotic prescription	14790	17146	16679	14746
Mean age [years] (SD)	43.58 (16.19)	43.87 (16.20)	44.37 (16.33)	44.53 (15.85)
Women *n* (%)	9416 (63.66)	11047 (64.43)	10706 (64.19)	9588 (65.02)
Arrhythmias *n* (%)	609 (4.12)	736 (4.29)	780 (4.68)	735 (4.98)
Pre-existing allergy *n* (%)	53 (0.36)	75 (0.44)	76 (0.46)	68 (0.46)
Acute exacerbation of chronic bronchitis (AECB)				
Patients with antibiotic prescription	18036	24651	23822	22124
Mean age [years] (SD)	68.42 (14.81)	69.33 (18.47)	69.42 (14.68)	70.20 (14.26)
Women *n* (%)	9276 (51.43)	12103 (49.10)	11770 (49.41)	10702 (48.37)
Arrhythmias *n* (%)	2901 (16.08)	4958 (20.11)	5039 (21.15)	5187 (23.45)
Pre-existing allergy *n* (%)	81 (0.45)	176 (0.71)	180 (0.76)	207 (0.94)

Data source—AOK PLUS Saxony

### Moxifloxacin

**Fig. 1:**
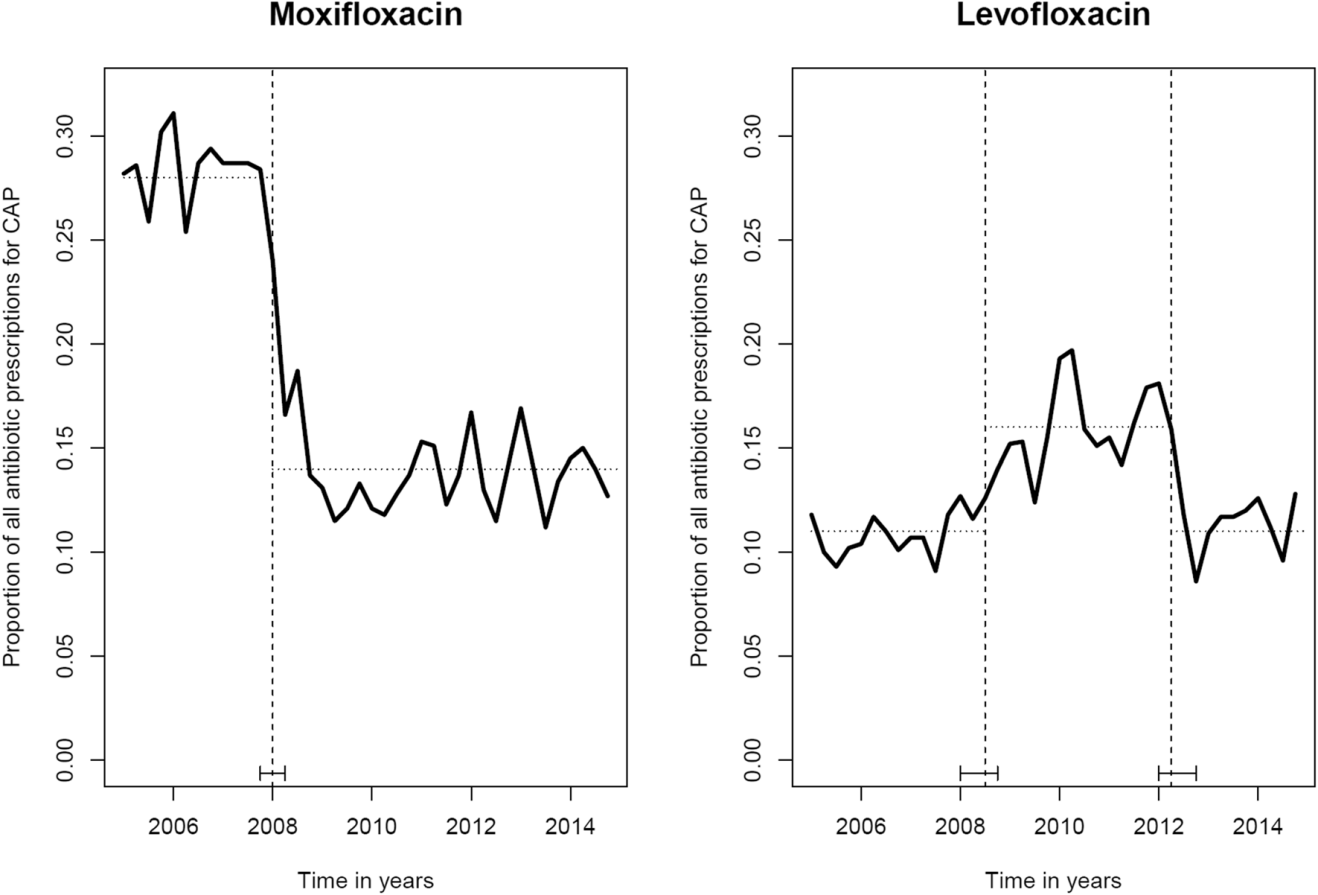
Display of the proportion of moxifloxacin and levofloxacin of all antibiotic prescriptions dispensed for diagnosed CAP in the time period from 2005 to 2014 (solid lines). Dotted lines represent the estimates’ breakpoints in the time series with corresponding confidence interval, considering the dates from the Dear Doctor Letters investigated. Data source—AOK PLUS Saxony

**Fig. 2:**
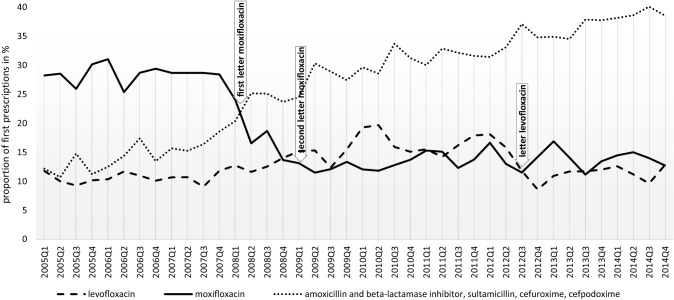
First prescriptions dispensed for diagnosed CAP in the time period from 2005 to 2014 including the published Dear Doctor Letters (moxifloxacin [02/2008, 01/2009], levofloxacin [09/2012]). Data source—AOK PLUS Saxony

The prescriptions of moxifloxacin for the treatment of CAP changed over time in the first quarter of 2008 (Fig. 1). At this point, a first Dear Doctor Letter [9] informed about new serious side effects and, as described in the introduction, the EMA restricted the use of oral moxifloxacin in the treatment of CAP, ABS and AECB [10]. As a result, the proportion of moxifloxacin prescribed was significantly reduced in the postintervention period 2009/2010 for the diagnoses analysed (Fig. 1, Supplement 1). As shown in Table 2, the individual risk ratio for being prescribed moxifloxacin decreased by 56% (95% CI 0.41–0.47; *p* < 0.001) for CAP, by 65% (95% CI 0.32–0.39; *p* < 0.001) for ABS and by 57% (95% CI 0.41–0.45; *p* < 0.001) for AECB. In 2009, when the second Dear Doctor Letter [11] was published in Germany, no changes in the prescribing behaviour over time could be detected for the diagnoses analysed (Fig. 1, Supplement 1). In addition, the warning for levofloxacin in September 2012 [12] had no significant impact on the moxifloxacin prescribing behaviour. The development in moxifloxacin first prescriptions for CAP is shown in Fig. 2 (for ABS, AECB see Supplement 2).

**Table 2:** Poisson regression with robust standard errors for different infection diseases and antibiotic first prescriptions dispensed; controlled for sex, age (linear), arrhythmia and pre-existing allergies against medications

	Dear Doctor Letter for moxifloxacin (02/2008)		Quarterly trend preintervention period I/2006–IV/2007 vs postintervention period I/2009–IV/2010			Dear Doctor Letter for levofloxacin (09/2012)		Quarterly trend preintervention period I/2010–IV/2011 vs postintervention period I/2013–IV/2014		
	Preintervention period *n* (%) 2006/2007	Postintervention period *n* (%) 2009/2010	RR	95% CI	*p* Value	Preintervention period *n* (%) 2010/2011	Postintervention period *n* (%) 2013/2014	RR	95% CI	*p* Value
Community-acquired pneumonia (CAP)										
Total antibiotic prescriptions	7189	8415				8256	8280			
Moxifloxacin	2068 (28.77)	1062 (12.62)	0.44	0.41–0.47	< 0.001	1110 (13.44)	1190 (14.37)	1.07	0.99–1.15	0.091
Levofloxacin	771 (10.72)	1365 (16.22)	1.51	1.39–1.64	< 0.001	1391 (16.85)	963 (11.63)	0.69	0.64–0.74	< 0.001
Amoxicillin/BLI, sultamicillin	383 (5.33)	811 (9.64)	1.79	1.59–2.02	< 0.001	902 (10.93)	1147 (13.85)	1.26	1.16–1.37	< 0.001
Cefuroxime	513 (7.13)	1279 (15.20)	2.12	1.93–2.34	< 0.001	1392 (16.86)	1681 (20.30)	1.21	1.13–1.29	< 0.001
Cefpodoxime	214 (2.98)	335 (3.98)	0.95	0.94–0.97	< 0.001	269 (3.26)	249 (3.01)	1.38	1.19–1.60	< 0.001
Other antibiotics	3240 (45.07)	3563 (42.34)	0.94	0.90–0.98	0.001	3192 (38.66)	3050 (36.84)	0.96	0.92–0.99	0.025
Acute bacterial sinusitis (ABS)										
Total antibiotic prescriptions	14790	17146				16679	14746			
Moxifloxacin	1225 (8.28)	501 (2.92)	0.35	0.32–0.39	< 0.001	548 (3.29)	459 (3.11)	0.94	0.84–1.07	0.384
Levofloxacin	520 (3.52)	666 (3.88)	1.10	0.98–1.23	0.091	712 (4.27)	541 (3.67)	0.86	0.77–0.96	0.007
Amoxicillin/BLI, sultamicillin	285 (1.92)	348 (2.03)	1.05	0.90–1.23	0.505	352 (2.11)	426 (2.89)	1.37	1.19–1.57	< 0.001
Cefuroxime	1010 (6.83)	2982 (17.39)	2.55	2.38–2.73	< 0.001	3419 (20.50)	4014 (27.22)	1.33	1.28–1.38	< 0.001
Cefpodoxime	364 (2.46)	690 (4.03)	1.34	1.13–1.58	0.001	643 (3.86)	545 (3.70)	0.92	0.78–1.09	0.332
Other antibiotics	11386 (76.98)	11959 (69.75)	0.91	0.89–0.92	< 0.001	11005 (65.98)	8761 (59.41)	0.90	0.89–0.92	< 0.001
Acute exacerbation of chronic bronchitis (AECB)										
Total antibiotic prescriptions	18036	24651				23822	22124			
Moxifloxacin	3185 (17.66)	1883 (7.64)	0.43	0.41–0.45	< 0.001	1910 (8.02)	1768 (7.99)	0.99	0.93–1.06	0.792
Levofloxacin	1409 (7.81)	3357 (13.62)	1.73	1.63–1.84	< 0.001	3126 (13.12)	2126 (9.61)	0.73	0.69–0.77	< 0.001
Amoxicillin/BLI, sultamicillin	611 (3.39)	1471 (5.97)	1.70	1.55–1.87	< 0.001	1517 (6.37)	1810 (8.18)	1.26	1.18–1.35	< 0.001
Cefuroxime	834 (4.62)	2780 (11.28)	2.43	2.25–2.62	< 0.001	3034 (12.74)	3827 (17.30)	1.35	1.30–1.42	< 0.001
Cefpodoxime	260 (1.44)	489 (1.98)	1.64	1.44–1.86	< 0.001	436 (1.83)	452 (2.04)	0.96	0.86–1.07	0.443
Other antibiotics	11737 (65.08)	14671 (59.51)	0.92	0.91–0.93	< 0.001	13799 (57.92)	12141 (54.88)	0.95	0.94–0.97	< 0.001

Data source—AOK PLUS Saxony

### Levofloxacin

Figure 1 and Supplement 1 show two structural breaks (2008, 2012) in the analysis of levofloxacin in the diagnosis of CAP and AECB and one break (2008) for ABS. The first shift occurred in 2008 shortly after the notification of the EMA [10] and the first Dear Doctor Letter for moxifloxacin [9]. This means that in the postintervention period 2009/2010 after the 2008 Dear Doctor Letter for moxifloxacin, levofloxacin was prescribed more frequently. As shown in Table 2, the individual risk ratio for being prescribed levofloxacin increased by 51% (95% CI 1.39–1.64; *p* < 0.001) for CAP, by 10% (95% CI 0.98–1.23; *p* = 0.091) for ABS and by 73% (95% CI 1.63–1.84; *p* < 0.001) for AECB. In 2012, the second shift in prescribing behaviour followed a safety warning regarding levofloxacin [12]. The restrictions affected CAP, ABS and AECB and complicated skin and soft tissue infections. As shown in Table 2, in the postintervention period 2013/2014 after the warning for levofloxacin in September 2012 [12], the risk ratio of a patient being prescribed levofloxacin decreased again by 31% (95% CI 0.64–0.74; *p* < 0.001) for CAP, by 14% (95% CI 0.77–0.96; *p* = 0.007) for ABS and by 27% (95% CI 0.69–0.77; *p* < 0.001) for AECB.

Additionally, the development in levofloxacin first prescriptions for CAP is shown in Fig. 2 (for ABS, AECB see Supplement 2).

### Cefuroxime, aminopenicillin/BLI or sultamicillin and cefpodoxime

Figure 2 shows a compensating effect to the declining prescriptions of moxifloxacin and levofloxacin as a result of the analysed Dear Doctor Letters for diagnosed CAP (for ABS, AECB see Supplement 2). The first prescriptions of alternatively recommended antibiotics increased over the whole study period, particularly for cefuroxime. The individual risk ratio for being prescribed cefuroxime increased by more than two times for the diagnoses analysed, especially in the postintervention period 2009/2010 (Table 2). Depending on the indication, the individual risk ratio for being prescribed aminopenicillin/BLI or sultamicillin increased by 5 up to 80% and decreased or increased for cefpodoxime (Table 2). The individual risk ratio for being prescribed other antibiotics decreased by around 4 up to 10% depending on the indication. A more detailed analysis of the other antibiotics was not part of the study.

### Arrhythmia and pre-existing allergy

The individual risk ratio of being prescribed a fluoroquinolone for CAP in combination with arrhythmia decreased by 7% for moxifloxacin after the warning in 2008 (95% CI 0.85–1.02; *p* = 0.135) and increased by 4% for levofloxacin after the warning in 2009 (95% CI 0.95–1.15; *p* = 0.401). The individual risk ratio of being prescribed a fluoroquinolone for ABS in combination with arrhythmia increased by 24% for moxifloxacin (95% CI 1.00–1.52; *p* = 0.041) and decreased by 6% for levofloxacin (95% CI 0.73–1.21; *p* = 0.637). The individual risk ratio of being prescribed a fluoroquinolone for AECB in combination with arrhythmia decreased by 3% for moxifloxacin (95% CI 0.90–1.04; *p* = 0.331) and by 5% for levofloxacin (95% CI 0.90–1.02; *p* = 0.150). In summary, the additionally diagnosed arrhythmia had no significant intended effect on the prescribing behaviour investigated. The additionally diagnosed pre-existing allergy had also no intended effect on the prescribing behaviour, details are presented in Supplement 3.

## Discussion

To our knowledge, this is the first study in Europe to explore the long-term effects of the safety warnings (Dear Doctor Letters) on fluoroquinolone use in community acquired respiratory tract infection. We carried out a population-based time series analysis based on > 200,000 first prescriptions over 10 years. These are the key results: Firstly, we found a significant and sustained reduction in the number of first prescriptions for moxifloxacin in 2008 and for levofloxacin in 2012 in relation to the documented diagnoses of CAP, ABS and AECB. What we did not find was a significant impact of the 2009 Dear Doctor Letter on moxifloxacin prescribing behaviour. In addition, we noticed a prescription-switch to other antibiotics like aminopenicillins, cephalosporins and partly levofloxacin. Secondly, we identified no prescribing behaviour change towards patients with an arrhythmia or pre-existing allergy.

The first safety warning in February 2008 [9] described potentially life-threatening side effects such as Stevens–Johnson syndrome or hepatic injuries related to moxifloxacin. The fact that guidelines valid at the time recommended alternative antibiotics suggests a prescription-switch to other antibiotics. An investigation from Italy [23] describes similar effects of safety warnings on antipsychotic drugs in dementia. After safety warnings in 2004 regarding risperidone and olanzapine, the use of both substances decreased whereas the use of quetiapine increased. Therefore, we conclude that the prescription-switch was a result of the Dear Doctor Letter as well. It should also be mentioned that we noticed a slight decrease in moxifloxacin prescriptions and an increase in other antibiotics even before the publication of the Dear Doctor Letter in 2008. We explain the early prescription-switch by the fact that the EMA evaluated the benefit/risk ratio of a moxifloxacin treatment already in 2007 [24]. The second safety warning for moxifloxacin in 2009 supported the recommendation of the first Dear Doctor Letter and in addition limited the indications to CAP, ABS and AECB. A supposed reason for the marginal reduction after the second safety warning could be the proximity in time to the first warning without important new aspects on side effects. Furthermore, we noticed a significant reduction of levofloxacin prescriptions after the Dear Doctor Letter in 2012, which like moxifloxacin limited the indication of levofloxacin to CAP, ABS and AECB. We speculate, that the two safety warnings on moxifloxacin had already sensitised prescribers to possible side effects and may have influenced the impact of the safety warning for levofloxacin. Fluoroquinolones were already known to cause side effects like arrhythmias at that time [25, 26]. In addition, several fluoroquinolones had already been withdrawn from the market due to a negative benefit-risk ratio [27]. Moreover, it should be added that there were no prescription restriction of moxifloxacin and levofloxacin by the health insurance company or any antimicrobial stewardship activities in the outpatient sector over the study period. In summary, the decreasing number of first prescriptions of moxifloxacin and levofloxacin and the observed structural breaks in the time series indicate that two safety warnings showed the intended impact on prescribing behaviour and one did not.

We also examined the impact of additionally documented arrhythmia or pre-existing allergy (in case of a penicillin allergy) on prescribing behaviour. We could find no significant influence on the prescribing behaviour in respect to arrhythmia or allergy over the whole study period, although the results of a recent systematic review and the meta-analysis [28] confirm the known fact that there is an association between the fluoroquinolone use and an increased risk of cardiac arrhythmia and cardiovascular mortality. Our findings concerning arrythmia and pre-existing allergy were confirmed by a German study [4]. The authors postulated that complicated contents of safety warnings like contraindicated co-medication facing for example QT interval-prolonging drugs seem to have significantly less influence on prescribing behaviour.

### Strengths and limitations

The strength of this study is the precise assignment of diagnosis and prescription data, the long period of time (40 quarters) analysed and the large number of prescriptions (184,134) investigated. With the analysis method by Bai and Perron [20], we were able to identify possible points of change within the time series. We applied a target-oriented method for data analysis, even without the 50 data points recommended to analyse an interrupted time series [29]. Additionally, to our knowledge, this is the first study in Europe to examine the impact of safety warnings for moxifloxacin and levofloxacin on prescribing behaviour. Despite that, our study has a few limitations. There is a possibility for classification bias with regards to the physician’s diagnosis. However, this does not affect our results as the focus of the study was on the physician’s prescribing behaviour. The number of cases for the additional diagnoses of arrhythmia and allergy was small, therefore the power was insufficient to validly examine the influence on prescribing behaviour. The missing structural break for the diagnosis ABS due to the 2012 Dear Doctor Letter for levofloxacin can be explained by the small number of first prescriptions. Furthermore, the question could be asked of how applicable our results are to other German federal states or to other European countries? Our study covers prescriptions of a large part of the Saxon population. However, there is no reason to believe that Saxony would differ from other German federal states. But caution should be used when generalising these results to other European countries.

### Additional aspects

Our study raises several questions: What are the causes for the remarkably high proportion of moxifloxacin first prescriptions before the safety warnings? What are the causes for the prescription switch mainly to cefuroxime after the safety warnings? National guidelines did not recommend moxifloxacin, levofloxacin or cefuroxime as first-line therapy for the diagnoses examined at the time the safety warnings were published [6, 7, 14]. Furthermore, should therapeutic alternatives be part of the safety warnings? The “Guidelines on good pharmacovigilance practices” (GVP) Module XV of the EMA [30] do not describe any specific requirements. Possibly the addition of therapeutic alternatives would support the intended impact of safety warnings and help in practical implementation. As French authors [31] or authors from the US [2] have already postulated, the lack of information on therapeutic alternatives can make it more difficult for physicians to rethink their prescribing behaviour.

## Conclusion

We identified significant changes in prescribing behaviour for two out of three Dear Doctor Letters investigated. Concerning patient safety, the results are encouraging. References on therapeutic alternatives were missing in the warnings examined but should be an obligatory part of any safety warning in future. Similarly, more importance should be attached to the adherence to national guideline recommendations. To further investigate the impact of Dear Doctor Letters on prescribing behaviour, the warning on fluoroquinolones published in April 2019 [32] should also be analysed.

## Electronic supplementary material

Below is the link to the electronic supplementary material.

Supplementary file1 (DOCX 220 KB)

Supplementary file2 (DOCX 169 KB)

Supplementary file3 (DOCX 17 KB)
