# Correction to: Impact of safety warnings for fluoroquinolones on prescribing behaviour. Results of a cohort study with outpatient routine data

**DOI:** 10.1007/s15010-021-01616-7

**Published:** 2021-05-17

**Authors:** Ulrike Georgi, Falko Tesch, Jochen Schmitt, Katja de With

**Affiliations:** 1 Pharmacy Service of Clinical Center, Flemmingstrasse 2, 09116 Chemnitz, Germany; 2 Center for Evidence-Based Healthcare, University Hospital and Medical Faculty Carl Gustav Carus, TU Dresden, Dresden, Germany; 3 Division of Infectious Diseases, University Hospital Carl Gustav Carus, TU Dresden, Dresden, Germany

## Correction to: Infection 10.1007/s15010-020-01549-7

The original version of this article unfortunately contained a mistake. The presentation of Fig. 2 was incorrect. The corrected Fig. 2 is given below.

**Fig. 2:**
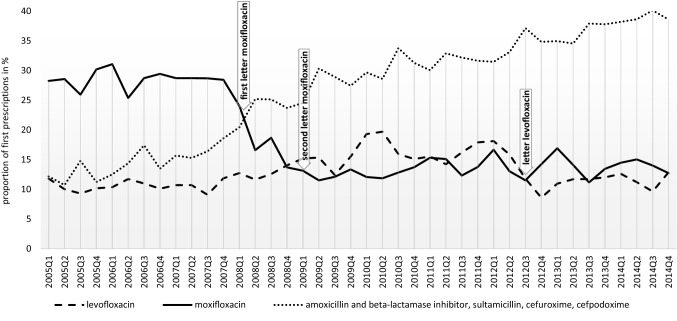
First prescriptions dispensed for diagnosed CAP in the time period from 2005 to 2014 including the published Dear Doctor Letters (moxifloxacin [02/2008, 01/2009], levofloxacin [09/2012]). Data source—AOK PLUS Saxony

The original article has been corrected.

