# Supplementary material for: Impact of safety warnings for fluoroquinolones on prescribing behaviour. Results of a cohort study with outpatient routine data
Source: Infection. 2020 Nov 30;49(3):447–55. doi: 10.1007/s15010-020-01549-7 (PMC8159769; doi:10.1007/s15010-020-01549-7)
Supplement: Supplementary file 1 — Supplementary file1 (DOCX 220 KB) [file 15010_2020_1549_MOESM1_ESM.docx]

**Impact of safety warnings for fluoroquinolones on prescribing behaviour. Results of a cohort study with outpatient routine data.**

Supplement 1

Display of the proportion of moxifloxacin and levofloxacin of all antibiotic prescriptions dispensed for diagnosed ABS or AECB in the time period from 2005 to 2014 (solid lines). Dotted lines represent the estimates’ breakpoints in the time series with corresponding confidence interval, considering the dates from the Dear Doctor Letters investigated. Data source – AOK PLUS Saxony.


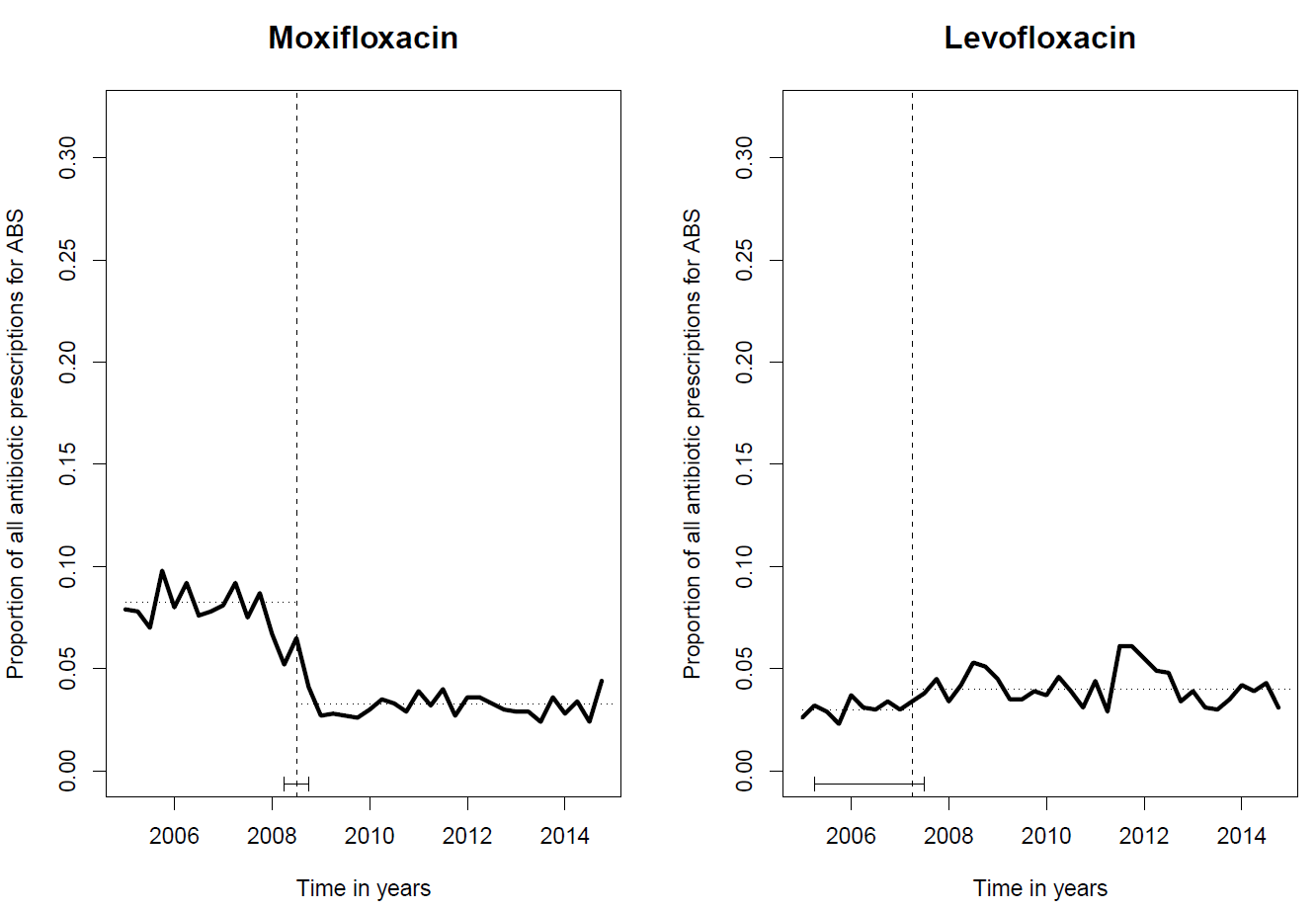


Diagnosis ABS


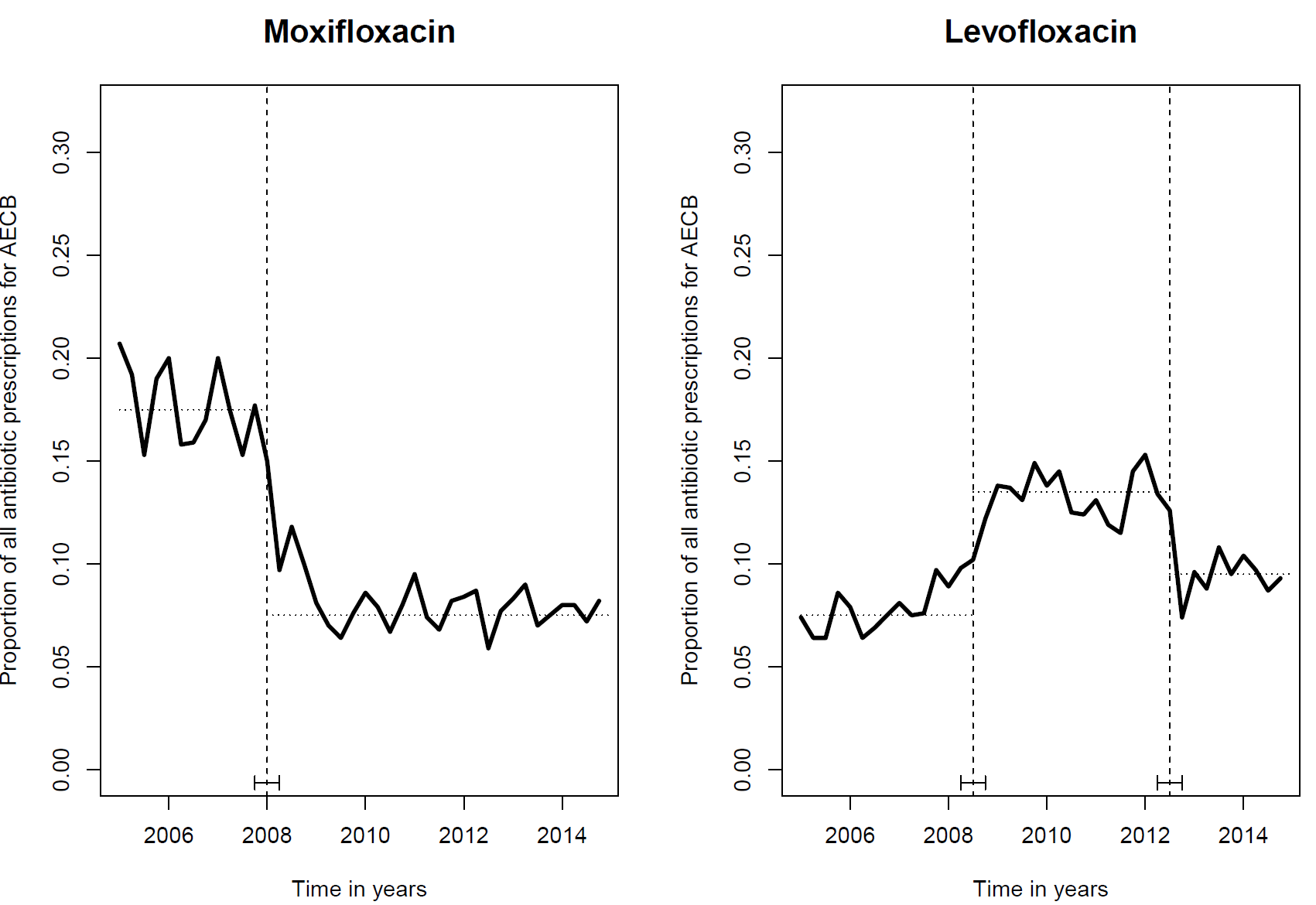


Diagnosis AECB
