# Supplementary material for: Impact of safety warnings for fluoroquinolones on prescribing behaviour. Results of a cohort study with outpatient routine data
Source: Infection. 2020 Nov 30;49(3):447–55. doi: 10.1007/s15010-020-01549-7 (PMC8159769; doi:10.1007/s15010-020-01549-7)
Supplement: Supplementary file 2 — Supplementary file2 (DOCX 169 KB) [file 15010_2020_1549_MOESM2_ESM.docx]

**Impact of safety warnings for fluoroquinolones on prescribing behaviour. Results of a cohort study with outpatient routine data.**

Supplement 2

First prescriptions dispensed for diagnosed ABS or AECB in the time period from 2005 to 2014 including the published Dear Doctor Letters (moxifloxacin [02/2008, 01/2009], levofloxacin [09/2012]). Data source – AOK PLUS Saxony.


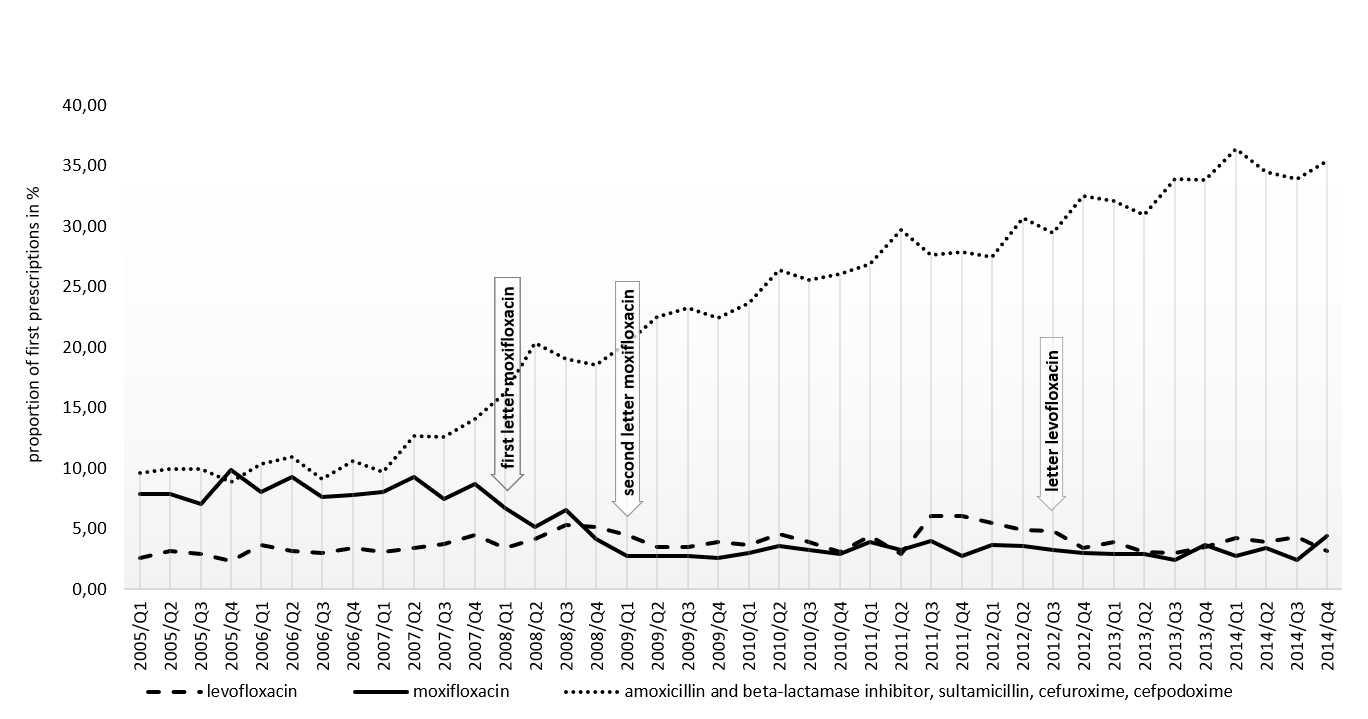


Diagnosis ABS


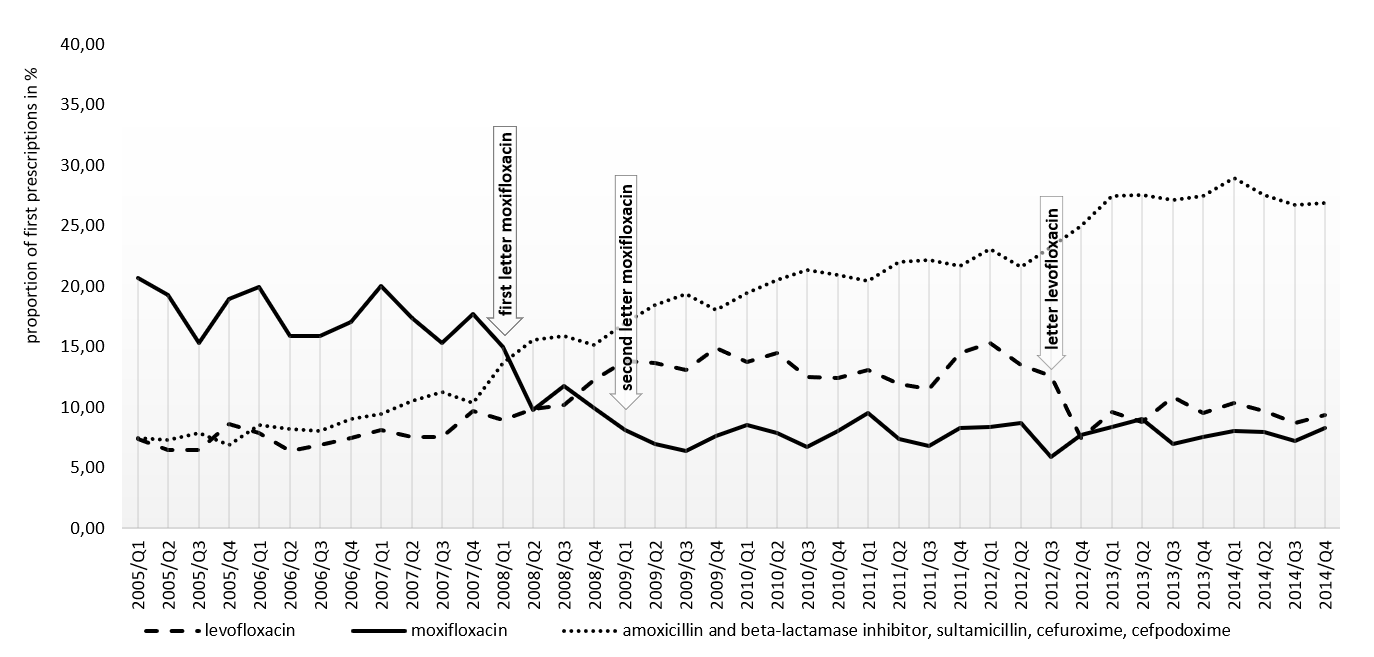


Diagnosis AECB
