# Supplementary material for: Impact of safety warnings for fluoroquinolones on prescribing behaviour. Results of a cohort study with outpatient routine data
Source: Infection. 2020 Nov 30;49(3):447–55. doi: 10.1007/s15010-020-01549-7 (PMC8159769; doi:10.1007/s15010-020-01549-7)
Supplement: Supplementary file 3 — Supplementary file3 (DOCX 17 KB) [file 15010_2020_1549_MOESM3_ESM.docx]

**Impact of safety warnings for fluoroquinolones on prescribing behaviour. Results of a cohort study with outpatient routine data.**

Supplement 3

Variables - sex, age (linear), arrhythmia and pre-existing allergies in the time period from 2005 to 2014. Data source – AOK PLUS Saxony.

|  |  | **Model moxifloxacin**  **2006/2007 to 2009/2010** | | | | | **Model levofloxacin**  **2010/2011 to 2013/2014** | | | | | |
| --- | --- | --- | --- | --- | --- | --- | --- | --- | --- | --- | --- | --- |
| Diagnosis | Variable | RR | 95 % CI | | P-Value | | RR | | 95 % CI | | P-Value | |
| **Community-acquired pneumonia (CAP)** | | | |  | |  | |  | |  | |  |
|  | Age (linear) | 1.00 | 1.00-1.00 | | 0.027 | | 1.00 | | 1.00-1.00 | | 0.015 | |
|  | Sex (ref. women) | 1.05 | 0.99-1.12 | | 0.098 | | 1.04 | | 0.96-1.12 | | 0.298 | |
|  | Arrhythmias | 0.93 | 0.85-1.02 | | 0.135 | | 1.04 | | 0.95-1.15 | | 0.401 | |
|  | Pre-existing allergy | 1.05 | 0.64-1.71 | | 0.851 | | 1.00 | | 0.60-1.65 | | 0.987 | |
| **Acute bacterial sinusitis (ABS)** | |  |  | |  | |  | |  | |  | |
|  | Age (linear) | 1.00 | 1.00-1.01 | | 0.008 | | 1.00 | | 1.00-1.01 | | 0.002 | |
|  | Sex (ref. women) | 0.96 | 0.87-1.06 | | 0.421 | | 0.98 | | 0.88-1.10 | | 0.787 | |
|  | Arrhythmias | 1.24 | 1.00-1.52 | | 0.041 | | 0.94 | | 0.73-1.21 | | 0.637 | |
|  | Pre-existing allergy | 1.34 | 0.72-2.50 | | 0.358 | | 0.68 | | 0.26-1.79 | | 0.433 | |
| **Acute exacerbation of chronic bronchitis (AECB)** | | | | | | | | | | | |  |
|  | Age (linear) | 1.00 | 1.00-1.00 | | <0.001 | | 1.00 | | 1.00-1.00 | | <0.001 | |
|  | Sex (ref. women) | 1.22 | 1.16-1.28 | | <0.001 | | 1.12 | | 1.06-1.18 | | <0.001 | |
|  | Arrhythmias | 0.97 | 0.90-1.04 | | 0.331 | | 0.95 | | 0.90-1.02 | | 0.150 | |
|  | Pre-existing allergy | 1.17 | 0.85-1.62 | | 0.331 | | 1.01 | | 0.76-1.34 | | 0.959 | |

Variables - sex, age (linear), arrhythmia and pre-existing allergies
